# European LeukemiaNet laboratory recommendations for the diagnosis and management of chronic myeloid leukemia

**DOI:** 10.1038/s41375-023-02048-y

**Published:** 2023-10-04

**Authors:** Nicholas C. P. Cross, Thomas Ernst, Susan Branford, Jean-Michel Cayuela, Michael Deininger, Alice Fabarius, Dennis Dong Hwan Kim, Katerina Machova Polakova, Jerald P. Radich, Rüdiger Hehlmann, Andreas Hochhaus, Jane F. Apperley, Simona Soverini

**Affiliations:** 1 Faculty of Medicine, University of Southampton, Southampton, UK; 2 Klinik für Innere Medizin II, Universitätsklinikum Jena, Jena, Germany; 3 Centre for Cancer Biology and SA Pathology, Adelaide, SA Australia; 4 Laboratory of Hematology, University Hospital Saint-Louis, AP-HP and EA3518, Université Paris Cité, Paris, France; 5 Huntsman Cancer Center Salt Lake City, Salt Lake City, UT USA; 6 III. Medizinische Klinik, Medizinische Fakultät Mannheim, Universität Heidelberg, Mannheim, Germany; 7 Department of Medical Oncology and Hematology, Princess Margaret Cancer Centre, University Health Network, University of Toronto, Toronto, Canada; 8 Institute of Hematology and Blood Transfusion, Prague, Czech Republic; 9 Fred Hutchinson Cancer Center, Seattle, WA USA; 10 ELN Foundation, Weinheim, Germany; 11 Centre for Haematology, Imperial College London, London, UK; 12 Department of Clinical Haematology, Imperial College Healthcare NHS Trust, London, UK; 13 Department of Medical and Surgical Sciences, Institute of Hematology “Lorenzo e Ariosto Seràgnoli”, University of Bologna, Bologna, Italy

**Keywords:** Oncogenesis, Myeloproliferative disease

## Abstract

From the laboratory perspective, effective management of patients with chronic myeloid leukemia (CML) requires accurate diagnosis, assessment of prognostic markers, sequential assessment of levels of residual disease and investigation of possible reasons for resistance, relapse or progression. Our scientific and clinical knowledge underpinning these requirements continues to evolve, as do laboratory methods and technologies. The European LeukemiaNet convened an expert panel to critically consider the current status of genetic laboratory approaches to help diagnose and manage CML patients. Our recommendations focus on current best practice and highlight the strengths and pitfalls of commonly used laboratory tests.

## Background

Chronic myeloid leukemia (CML) is characterized by the Philadelphia (Ph) chromosome, described in 1960 as the first recurrent chromosome abnormality associated with a human malignancy [1]. The Ph chromosome, or more correctly the der22,t(9;22)(q34;q11), is the smaller derivative of a somatically acquired reciprocal translocation between chromosomes 9 and 22 [2], which leads to fusion of the Breakpoint Cluster Region (*BCR*) gene at 22q11 and the Abelson Proto-oncogene 1 Nonreceptor Tyrosine Kinase (*ABL1*) gene at 9q34 [3]. The resulting *BCR::ABL1* fusion, located on the Ph chromosome, encodes a chimeric BCR::ABL1 protein with deregulated tyrosine kinase activity that is the primary driver of the pathogenesis of CML [4]. Understanding the mechanism by which BCR::ABL1 leads to myeloproliferation focused subsequent research on the selective inhibition of its enzymatic activity, with the first-generation tyrosine kinase inhibitor (TKI), imatinib, developed partly through a process of rational drug design [5]. Following the International Randomized Study of Interferon and STI571 (IRIS trial), imatinib was approved as first-line treatment for newly diagnosed CML patients [6] and, along with second generation (2G; nilotinib, dasatinib, bosutinib), third generation (3G; ponatinib) and now fourth generation (4G; asciminib) TKIs, has resulted in a near-normal life expectancy for most CML patients in the developed world [7, 8]. In many low- and middle-income countries, however, response rates and survival are often suboptimal due to a combination of factors [9].

Treatment goals for CML have advanced well beyond considerations of survival to molecular assessments of the depth and stability of remission, and the possibility of attaining treatment-free remission (TFR) [10]. Despite the enormous progress in CML, the depth of responses for individual patients is heterogeneous. Some chronic phase (CP) CML patients with high-risk features, e.g., with high EUTOS Long-Term Survival Score (ELTS) or high-risk additional cytogenetic abnormalities (ACA), remain at significant risk for CML-related death despite currently available drug therapies. Furthermore, some patients present with refractory disease or develop secondary resistance, often associated with the acquisition of single nucleotide variants in the *BCR::ABL1* tyrosine kinase domain (TKD) that result in impairment of drug binding. In some cases, poor responses are associated with progression from CP towards an aggressive and usually terminal acute leukemia, known as blast crisis or blastic phase (BP), which may be of myeloid, lymphoid or mixed phenotype [11].

### General laboratory recommendations:

- All tests for which the results are used for clinical management should be conducted in appropriately accredited laboratories, e.g., to ISO15189.2022, and fully validated before clinical use.
- Testing laboratories should participate in appropriate external quality assurance (EQA) schemes.

## Diagnostic work up and pre-treatment tests

Since 2008, CML has been defined as *BCR::ABL1* positive disease only. Diagnosis of CML therefore requires the detection of the t(9;22)(q34;q11) and/or *BCR::ABL1* in the appropriate clinical and laboratory setting. Cases previously considered as *BCR::ABL1*-negative CML should be classified as another myeloid neoplasm depending on their hematological and molecular features [11, 12].

Historically, cytogenetic analysis of peripheral blood or bone marrow-derived cells played an essential role in the initial diagnosis of CML by direct detection of the t(9;22)(q34;q11), but many centres nowadays routinely use fluorescence in situ hybridisation (FISH) to detect juxtaposition of the *BCR* and *ABL1* genes in interphase cells, and/or reverse transcription polymerase chain reaction (RT-PCR) to detect *BCR::ABL1* mRNA as first line diagnostic tools in patients with a clinical suspicion of CML, with cytogenetic follow up for confirmation and detection of ACAs. Used in isolation, all three tests have limitations that need to be considered.

### Cytogenetics

At presentation, up to 85–90% of CML cases have a t(9;22)(q34;q11) on cytogenetic analysis and thus have a standard Ph chromosome, sometimes described as 22q- or der22. A minimum of 20 metaphases should be analysed (ideally 25) to ensure detection of ACAs. In most patients ≥95% of cells are found to be Ph-positive, which reflects the marked expansion of clonal myeloid progenitor cells induced by *BCR::ABL1*. A bone marrow aspirate is preferred for cytogenetic analysis and can be performed on the same sample taken for morphological analysis to diagnose disease phase, but a karyotype may be successfully obtained from peripheral blood in many cases at diagnosis (but not at follow up when bone marrow is required). In 5-10% of cases the karyotype shows a variant translocation that typically involves either or both 9q34 and 22q11 plus one or more additional chromosomes. The remaining 1-5% of cases have a cryptic *BCR::ABL1* fusion without any cytogenetically-visible involvement of chromosomes 9 or 22. These cases typically arise by small double recombination events that insert *ABL1* into *BCR* [13, 14] and thus they cannot be diagnosed as CML by conventional cytogenetics.

Around 7% of CP cases present with ACAs in the CML clone that are unrelated to the t(9;22). ACAs are also acquired with higher frequency during the course of the disease and are much more common in BP [15]. Clonal chromosome abnormalities may also be found in Ph-negative cells. If detected at diagnosis, major-route ACAs [+Ph, +8, i(17q), +19] [16] are associated with longer times to achieve complete cytogenetic remission (CCR) and major molecular response (MMR) as well as shorter progression-free and overall survival, at least for patients treated with imatinib [17]. Consequently, they have been termed high-risk ACAs. More recent analyses have indicated that +21, +17, -7/7q-, 3q26.2, 11q23 rearrangements and complex karyotypes should also be considered as high-risk ACAs, while other changes such as -Y in men do not affect prognosis [18, 19]. Variant translocations and cryptic *BCR::ABL1* rearrangements are not thought to be of any prognostic significance [17, 20, 21]. The presence of clonal chromosome abnormalities in Ph-negative cells (which are more commonly seen in patients undergoing treatment than at diagnosis, and occasionally may be transient in nature) has been associated with hematological toxicity of TKI therapy [22], but do not have a significant impact on the overall prognosis, with the exception of -7/del(7q) which has been associated with inferior response in some cases as well as dysplasia, myelodysplastic syndrome (MDS) and progression to acute myeloid leukemia (AML) [23–25]. Cytogenetic results should be reported according to the current version of International System for Human Cytogenomic Nomenclature (ISCN) [26] and use recommended nomenclature for fusion genes [27].

### Fluorescence in situ hybridization (FISH)

FISH using commercially available probes efficiently detects essentially all types of *BCR::ABL1* rearrangement irrespective of any cytogenetic or molecular nuances, but it does not detect ACAs. Interphase FISH is widely employed as an initial screening tool for CML, with the analysis usually being performed on peripheral blood leukocytes. Interphase FISH on blood granulocytes may also be useful to differentiate between de novo lymphoid BP-CML (granulocytes positive for *BCR::ABL1*) and de novo *BCR::ABL1*-positive acute lymphoblastic leukemia (ALL; granulocytes negative for *BCR::ABL1*). Metaphase FISH may be useful to confirm the presence and location of *BCR::ABL1* in cases with variant translocations or cryptic rearrangements. FISH negative, *BCR::ABL1* positive patients may theoretically arise due to very small double recombination events but these are exceedingly rare and, to our knowledge, only a single fully validated FISH negative CML case has been described [28].

### Qualitative RT-PCR and *BCR::ABL1* isoforms

The t(9;22) genomic breakpoints are widely dispersed, particularly at the *ABL1* locus, but splicing of the primary transcript gives rise to a limited number of *BCR::ABL1* mRNA isoforms [29]. The two most common are referred to as e13a2 (*BCR* exon 13 spliced to *ABL1* exon 2) and e14a2 (*BCR* exon 14 spliced to *ABL1* exon 2). Historically, these two chimeric mRNAs were referred to as b2a2 and b3a2, with b2 (*BCR* exon 13) and b3 (*BCR* exon 14) corresponding to the second and third exons within the classically-defined major breakpoint cluster region (M-BCR) [30]. Both e13a2 and e14a2 encode a 210 kDa BCR::ABL1 protein (p210), but e14a2 BCR::ABL1 is 25 amino acids larger than e13a2, due to the additional amino acids encoded by *BCR* exon 14. Collectively, e13a2 and e14a2 *BCR::ABL1* account for 98% of CML cases, with the majority of these expressing e14a2. Around 10% of cases express both isoforms. A large international survey of CML cases found small but significant differences in the relative prevalence of the two fusions in relation to age and gender, with the prevalence of e13a2 being higher in males compared to females and lower in the elderly [31]. In addition, several studies have shown that patients expressing e13a2 *BCR::ABL1* have inferior molecular responses compared to those expressing e14a2 [32–34], an observation that is discussed in more detail below.

**Fig. 1:**
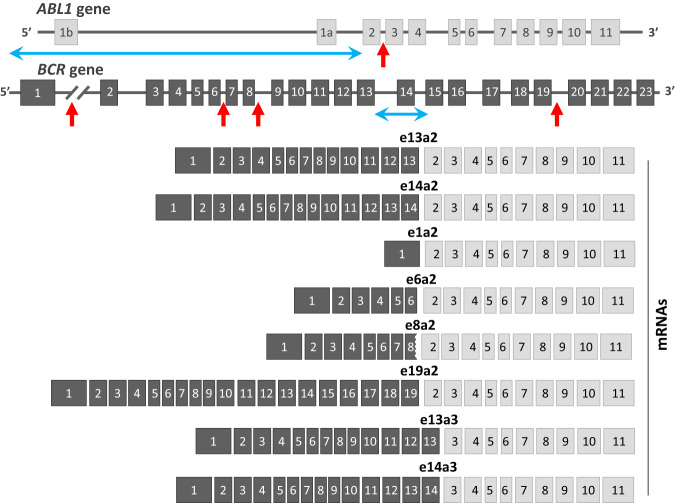
*BCR::ABL1* fusions. The genomic configurations of the *ABL1* (11 exons) and *BCR* genes (23 exons) are shown at the top. In the great majority of CML patients the genomic breakpoints fall in the regions indicated by the two horizontal double blue arrows, i.e. 5’ of *ABL1* exon 2 and between exons 13 and 15 of *BCR*, giving rise to e13a2 and/or e14a2 *BCR::ABL1* mRNA fusions. The approximate positions of recurrent variant breakpoints are indicated by the red vertical arrows giving rise to mRNA fusions that involve different *BCR* exons and/or *ABL1* exon 3. Some of these atypical variants are illustrated (e1a2, e6a2, e8a2, e19a2, e13a3, e14a3) but other variants are seen in occasional cases. *BCR* exon 8 is out of frame with *ABL1* exon 2; e8a2 fusions typically break within *BCR* exon 8 or additional intron-derived sequences are retained to maintain the reading frame. Diagrams are illustrative and not to scale.

Around 2% of CML patients express atypical *BCR::ABL1* fusions that arise from *BCR* breakpoints outside the M-BCR or downstream of *ABL1* exon 2. The most common are e1a2, e6a2, e8a2, e19a2, e13a3, and e14a3, i.e. mRNA fusions of *BCR* exons 1, 6, 8, 19 to *ABL1* exon 2, or *BCR* exons 13 or 14 fused to *ABL1* exon 3 (Fig. 1). Other very rare fusions have also been described, including patient-specific variants with unique fusion exons and/or incorporation of intronic sequence or exons from other genes as a result of complex genomic rearrangements [31]. Atypical *BCR::ABL1* fusions encode different size proteins, e.g., e1a2 and e19a2 mRNA fusions encode 190 kDa (p190) and 230 kDa (p230) BCR::ABL1, respectively.

The reciprocal product of the t(9;22), *ABL1::BCR*, is expressed in approximately 70% of CML patients but is not itself believed to play a role in the disease process [35, 36]. However, absence of *ABL1::BCR* expression is linked to deletions at the genomic breakpoints on the der9,t(9;22)(q34;q11) [37]. Largely of historical interest, these deletions are associated with inferior outcomes in patients treated with interferon-α, while TKIs mostly override the adverse effect [38, 39].

### Initial identification of CML patients

For centres that use reverse transcription polymerase chain reaction (RT-PCR) based methods as the primary or sole means to screen for *BCR::ABL1* in cases with suspected CML, it is essential that the methodology detects both typical and atypical *BCR::ABL1* variants. Screening only for transcripts that encode p210 or p210 plus p190 BCR::ABL1 is poor practice and may result in up to 2% of *bone fide* CML cases being misdiagnosed as another disorder. If a limited transcript screen is performed it is essential that the clinical report clearly states that atypical *BCR::ABL1* fusions would not have been detected, and that a diagnosis of CML is not excluded with certainty.

For centres that use cytogenetics and/or FISH as the primary screen, the *BCR::ABL1* transcript type in positive cases should be determined prior to starting treatment to facilitate accurate assessment of measurable residual disease (MRD) on therapy. Although the exact transcript type should ideally be identified for all patients, many groups use reverse transcriptase quantitative PCR (RT-qPCR) to assess levels of e13a2 and/or e14a2 mRNA in cases prior to treatment and only search for atypical fusions in the small minority of CML cases for whom *BCR::ABL1* mRNA is not detected, or found at unexpectedly low levels (e.g. <1% on the International Scale). Low levels of *BCR::ABL1* mRNA at diagnosis may also signal the presence of a co-existing *BCR::ABL1*-negative hematological neoplasm. Quantitative assessment of *BCR::ABL1* mRNA prior to treatment may also be useful to help interpret initial MRD results, e.g. after 3 months on therapy [40–42], as discussed below.

Identification of atypical *BCR::ABL1* variants may be challenging, and there are no CE-marked or Food and Drug Administration (FDA)-approved kits or systems that can detect most rare isoforms. Laboratory-developed tests (LDTs) have been described that use multiplex RT-PCR [43] followed, where appropriate, by sequence analysis. Two step, nested RT-PCR should generally not be used in patients with a high burden of disease due to the potential for artefacts or amplification of very low level, clinically insignificant splice variants. Looking forward, it is likely that genomic approaches such as whole transcriptome sequencing will be used increasingly on a routine basis to identify *BCR::ABL1* isoforms as well as other fusion genes.

### Features of patients with atypical *BCR::ABL1* fusions

Several case reports have associated individual atypical *BCR::ABL1* fusions with an unusually aggressive or benign clinical course [29], but these are likely subject to substantial ascertainment or publication bias. In general, atypical variants are not considered to be markers of prognosis, although systematically collected outcome data is lacking due to their rarity and exclusion from many clinical trials. An exception may be the e1a2 fusion which encodes p190 *BCR::ABL1*, the predominant isoform in *BCR::ABL1*-positive ALL but also seen in 1% of CML cases. This isoform was originally associated with a phenotype intermediate between CML and chronic myelomonocytic leukemia (CMML) [29], and more recent data has indicated a relatively poor outcome, possibly associated with mutations in epigenetic modifiers genes [44–46]. Other studies, however, have not confirmed this finding [47] and consequently we consider that therapeutic decisions should not be influenced by *BCR::ABL1* transcript type. Nevertheless, identification of cases who express an atypical *BCR::ABL1* variant is required prior to treatment to enable appropriate follow up analysis.

**Fig. 2:**
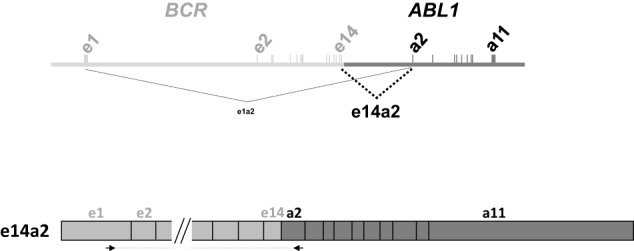
Low level expression of e1a2 *BCR::ABL1* in e13a2/e14a2 cases. Top panel: illustration of low level e1a2 mRNA transcripts generated by alternative splicing in a patient expressing high level e14a2 *BCR::ABL1*. Bottom panel: example showing how an apparent e1a2 signal may be generated from intact e14a2 mRNA (depending on the positions of primers, probe, cDNA quality and amplification kinetics). Misinterpretation of low-level *BCR::ABL1* products may lead to incorrect assignment of transcript *BCR::ABL1* type, and incorrect molecular follow up on treatment.

It is important to note that the *BCR::ABL1* isoform in any given patient is stable over time since it is determined by the position of the t(9;22) genomic breakpoints. However, the sensitivity and specificity of RT-qPCR may occasionally cause some confusion. For example, rearrangements that produce e13a2 and/or e14a2 *BCR::ABL1* have the capability to generate e1a2 mRNA and other isoforms by alternative splicing (Fig. 2). Indeed, very low levels of e1a2 transcripts can be detected in most cases of CML prior to treatment [48] but they are generally believed to be of no clinical significance, although one study did find that co-expression of p210 and p190 mRNA was associated with an adverse prognosis [49]. Such cases should not be considered as p190 CML since the level of e1a2 expression is typically >1000x lower than that of the predominant p210 isoform, and *bone fide* clonal evolution from p210 to p190 is exceedingly rare. P190 or other rare variant CML should only be diagnosed when expression of the atypical *BCR::ABL1* isoform is at a level consistent with the burden of pre-treatment disease, i.e. comparable to that seen in standard e13a2/e14a2 cases. Similarly, as mentioned above, patients with atypical *BCR::ABL1* fusions such as e19a2 may express low levels of e13a2 and/or e14a2 *BCR::ABL1* mRNA and thus the presence of an atypical fusion may be missed [50].

### Recommendations:

- Cytogenetics along with FISH and/or RT-PCR should be used in all cases to confirm a diagnosis of CML. The limitations of each approach as standalone tests need to be understood and, where appropriate, included in clinical reports.
- Cytogenetic testing should include a screen for ACAs at diagnosis.
- *BCR::ABL1* mRNA transcript type should be determined for all cases prior to treatment to enable appropriate follow up.
- The possibility of a rare *BCR::ABL1* variant should be excluded. If testing for rare variants is not available, the diagnostic report should clearly state that the presence of a *BCR::ABL1* remains a possibility and that further testing in an appropriate reference laboratory should be performed.

### Genomics and additional genetic abnormalities

Once a diagnosis of CML has been made, additional laboratory investigations may help to define prognosis (Table 1). Multiple studies have reported results from mutational analysis of a broad range of cancer-associated genes in CML using targeted next generation sequencing (NGS) panels, whole exome sequencing (WES) or whole genome sequencing (WGS), as reviewed in detail elsewhere [51, 52]. These studies are heterogeneous with regard to patient selection, genes tested, methodology employed and criteria for calling relevant mutations but nevertheless a number of important conclusions have emerged that build on previous studies using Sanger sequencing, DNA arrays and cytogenetics: (i) a wide range of somatically mutated genes are found in CML, most commonly in BP but also in CP; (ii) distinct mutational profiles are associated with myeloid and lymphoid BP; (iii) mutations in genes associated with age-related clonal hematopoiesis (CH) may precede the acquisition of *BCR::ABL1* or be acquired subsequent to *BCR::ABL1*; (iv) some studies have found that detection of *ASXL1* mutations in CP is associated with inferior response to TKI treatment.

**Table 1:** Recommended tests for diagnostic workup of CML patients.

	ROUTINE DIAGNOSTIC WORKUP	EXPERIMENTAL/CLINICAL TRIALS^c^
**Interphase FISH**	• **Recommended for initial screening**^**a**^ Pros: Picks up all *BCR::ABL1* rearrangements irrespective of breakpoint. Usually performed on PB. May be used as primary screen for *BCR::ABL1* or to investigate cases that show discrepant results between cytogenetics and RT-PCR. Cons: does not detect ACAs or identify transcript type therefore positive cases need to be followed up by both cytogenetics and RT-PCR.	• **Acceptable for initial screening**
**Qualitative RT-PCR**	• **Recommended for initial screening**^**a**^ • **Strongly recommended for determining** ***BCR::ABL1*** **transcript type in all confirmed CML patients** • **Mandatory to detect atypical** ***BCR::ABL1*** **variants**^**b**^ Pros: Only routine technique to determine exact *BCR::ABL1* transcript type^b^. Usually performed on PB. Cons:. No commercial test available to detect most atypical *BCR::ABL1* variants, but essential to cover atypical variants if used as a primary screen. Sequence confirmation may be required. Cannot identify ACAs therefore positive cases need to be followed up by cytogenetics. Nested RT-PCR should not generally be used due to the risk of artefacts and contamination.	• **Mandatory**
**Cytogenetics**	• **Mandatory for all CML cases** Pros: Only routine technique that can detect prognostically significant ACAs. Can usually be performed on PB but may require BM. May be performed after initial screening by FISH or RT-PCR. Metaphase FISH may be useful to investigate variant translocations. Cons: Up to 5% of CML patients have a normal karyotype therefore not recommended in isolation as an initial screening tool. Positive cases need to be followed up by RT-PCR to determine *BCR::ABL1* transcript type.	• **Mandatory**
**Quantitative RT-qPCR**	• **Not generally recommended** Pros: may provide additional prognostic information by providing baseline to determine early response kinetics. May be used as an initial screen for CML cases identified by FISH or cytogenetics to identify (by exclusion) those who need investigation for atypical *BCR::ABL1* variants. Cons: approach to determine early response kinetics not standardized.	• **Strongly recommended** *GUSB* or *BCR* recommended as reference genes in preference to *ABL1* to determine early response kinetics within the first 1-3 months
**NGS panel for myeloid and lymphoid genes**	• **Not recommended for CP** • **Suggested for** ***de novo*** **BP** Pros: May provide additional prognostic information and identify targets for therapy Cons: Very limited clinical actionability, even in BC	• **Strongly recommended for CP and** ***de novo*** **BP** *ASXL1* mutations associated with adverse prognosis in some contexts but further studies required to define actionability
***BCR::ABL1*** **TKD mutations**	• **Not recommended for either CP or** ***de novo*** **BP** Mutations very unlikely to be detected prior to TKI therapy	• **Not generally recommended (but should be considered)**

^a^FISH and qualitative RT-PCR may be considered as alternative approaches for initial screening, i.e. initial identification of CML patients.

^b^RNAseq may be a better alternative to qualitative RT-PCR but is not widely available.

^c^Depending on trial design and objectives.

**Fig. 3:**
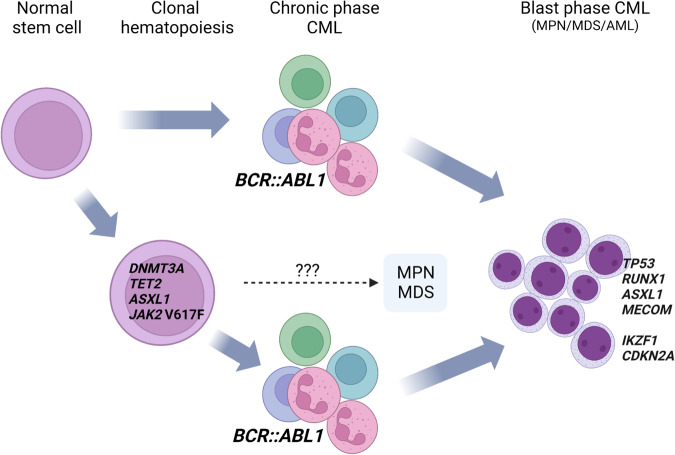
Model of two pathways to CML. In most cases, a normal hematopoietic stem cell acquires the *BCR::ABL1* fusion leading to the development of CP CML (top pathway). Acquisition of additional mutations and epigenetic changes eventually precipitate a block in differentiation and transition to BP. Some of the more commonly mutated genes at BP are indicated: *TP53*, *RUNX1*, *ASXL1* and *MECOM* are associated with myeloid BP; *IKZF1* and *CDKN2A/B* are associated with lymphoid BP. In some cases, *BCR::ABL1* is acquired on a background of CH (either as a CH subclone or independently of the CH clone), for example CH driven by mutations in *DNMT3A*, *TET2, ASXL1* or *JAK2* (bottom pathway). These pre-existing mutations remain detectable during remission on TKI therapy. In contrast, for the top pathway any additional mutations found pre-treatment become undetectable at remission. The dotted line indicates a potential route to transformation from the CH clone (which may also develop ACAs) to a *BCR::ABL1*-negative myeloid neoplasm such as MPN or MDS. Figure created using BioRender.

Overall, variants in cancer-associated genes (not including *BCR::ABL1* TKD mutations) have been described in 20-30% of CML patients in CP, but it is important to note that most published studies are retrospective and focused on patients who subsequently relapsed or progressed to BP. Prospective studies are needed to determine the true frequency of genetic variants in CP, and their clinical significance. Nevertheless, *ASXL1* has emerged as the most common somatically-mutated gene in CP [51, 52], with abnormalities seen in about 10% of adult CML patients, although the prevalence in children and young adults appears to be significantly higher [53]. Other recurrent abnormalities in CP include mutations in *RUNX1* (2%), *TET2* (2%), and *DNMT3A* (2%), and mutations or deletions of *IKZF1* (4%). Of note, *DNMT3A*, *TET2* and *ASXL1* mutations are commonly associated with CH and in some cases may precede the acquisition of *BCR::ABL1* [54]. In these patients, the mutations typically persist despite effective suppression of the CML clone by TKI therapy (Fig. 3). Nevertheless, some studies have found that *ASXL1* mutations [55, 56] or overall somatic mutation burden [57] prior to treatment are associated with poor outcomes.

The clinical actionability of mutations in *ASXL1* or other genes, however, has not been established and thus routine screening by myeloid NGS panel analysis or other approaches at presentation in CP is generally considered to be research rather a routine diagnostic requirement. In the future, however, once the clinical significance of these abnormalities is better understood it is expected that routine targeted screening of CML patients for somatic mutations prior to treatment will become more widespread.

As for constitutional genetics, a specific *HMGCLL1* haplotype and polymorphic variation at *BIM*, *ASXL1* and killer immunoglobulin-like receptor (KIR) loci have all been associated with response to TKI therapy or outcome after treatment cessation [58–63] but in the absence of clear clinical actionability and systematic validation, none are yet incorporated into routine risk stratification.

### Genomic abnormalities in BP

The great majority of patients who present with, or progress to, BP have additional somatic mutations in cancer-associated genes, with *ASXL1*, *RUNX1, BCOR/BCORL1* and *IKZF1* each being seen in 15-20% of cases [51, 52, 64]. *ASXL1* and *TP53* mutations are usually associated with myeloid BP, whereas *IKZF1* and *CDKN2A/B* mutations/deletions are associated with lymphoid BP [65, 66]. Mutations in a wide range of other genes have been reported that overlap with those seen AML and ALL, although mutations in *SETD1B* and *UBE2A* may be relatively specific to CML-BP. Although mutational patterns have been linked to prognosis, potentially targetable abnormalities are uncommon, e.g., *IDH1/2* and *NRAS* mutations in 5% of cases and *FLT3* mutations in very rare cases [66]. In addition, structural rearrangements are often acquired at BP, at least some of which are expected to be drivers of transformation. Some of these are associated with AML and have been known for many years to occur in myeloid BP by cytogenetic analysis e.g., *CBFB::MYH11* and rearrangements that target the *MECOM* locus. More recently, whole transcriptome sequencing (WTS) or targeted RNA sequencing has revealed that acquired fusion genes are more common than previously thought, with some targeting well known cancer genes such as *RUNX1*, but also others of uncertain pathogenicity [57, 67].

### Technical aspects of NGS

From a technical perspective, there are several points to consider in relation to somatic mutation screening. For example, the methodology (i.e., targeted NGS panel sequencing, WES or WGS), the limit of detection (LoD; linked in part to the depth of sequencing), the sample type (total leukocytes or sorted cell fractions) and the value of including germline samples in the analysis. Currently, most routine NGS analysis for hematological neoplasms is undertaken using targeted panels with an LoD of 3–5% variant allele frequency (VAF; number of mutant alleles/number of mutant plus wild type alleles x 100), a level corresponding to the technical sensitivity rather than a clinically-validated cut off. Error-corrected NGS can achieve better sensitivity (LoD ≤0.5%) but in the context of CML there is no evidence that the detection of low-level mutations is of any clinical benefit. Analysis of germline samples is typically limited to investigation of specific germline predisposition syndromes.

An ideal CML panel should cover both myeloid and lymphoid targets [51], for example including but not limited to *RUNX1*, *ASXL1*, *BCOR*, *BCORL1*, *TP53*, *IZKF1* [68], and there may be some advantages in screening RNA rather than DNA [69]. It is important to note that the NM_015338.6:*ASXL1* c.1934dup;p.Gly646Trpfs*12 (*ASXL1* c.1934dup) variant is widely seen in myeloid neoplasms, including CML, but it also occurs as a common technical artefact by PCR replicative slippage [70]. The VAFs of artefactual calls for *ASXL1* c.1934dup are highly dependent on the methodology used but may exceed 10%. It is essential that any laboratory reporting this variant establishes the background call rate with appropriate control samples (which is complicated by the fact that *ASXL1* variants are associated with CH) and only reports variants that are considered to have a high probability of being real, as described [71]. Detailed guidelines for diagnostic validation of NGS panels for clinical use have been described and should be followed [72, 73]. Reporting of variants should use Human Genome Variation Society (HGVS) nomenclature (https://varnomen.hgvs.org/) and include an assessment of relevance and pathogenicity as well as the variant allele frequency. Accurately ascribing pathogenicity to somatic variants (e.g. oncogenic/strong clinical significance, likely oncogenic/potential clinical significance, uncertain significance, likely benign, benign) is a complex process but structured classification guidelines continue to improve and should be employed [74–76]. Care should be taken in the interpretation of variants detected in samples with reduced *BCR::ABL1* levels since somatic variants could be derived from non-leukemia cells and represent CH or occasionally another disorder. Finally, the possibility of a likely irrelevant germline variant should be considered and potentially excluded when the VAF is close to 50% or 100%.

### Future perspective

RNA studies have revealed additional potentially important markers that may be incorporated into future diagnostic algorithms such as gene expression signatures predictive of resistance and/or progression [77, 78] and comprehensive detection of fusion genes including Ph-associated rearrangements that have been linked to adverse prognosis [67]. Single cell genomics has the potential to refine prognostication by linking specific mutations with cellular identity [79], whilst long read WGS may provide a comprehensive assessment of the somatic landscape of CML. With the accumulation of a wider evidence base, we anticipate that both DNA-based and RNA-based genomic tests will become integral to risk classification in CML [51] but for the time being these approaches remain firmly within the realms of research.

### Recommendations:

- Gene panel analysis pretreatment, including *ASXL1* mutation screening, is not currently recommended for routine clinical management but should be performed in investigational studies.
- NGS panel analysis for patients who present in, or progress to, BP is recommended to identify potential targets for treatment in addition to *BCR::ABL1*.

## Molecular monitoring of measurable residual disease

Sequential molecular monitoring of CML patients by reverse transcription quantitative polymerase chain reaction (RT-qPCR) has been established for many years using either LDTs or commercial kits/systems to assess the depth of clinical response to TKIs or identify early relapse after stem cell transplantation [80]. Levels of *BCR::ABL1* mRNA in peripheral blood leukocytes from patients on treatment serve as a surrogate for disease burden, and strongly correlate with outcome. Reverse transcription digital PCR (RT-dPCR) is a valid alternative to RT-qPCR and, when optimised, can provide more accurate results across a broad spectrum of disease levels but may be of particular value at very low levels of disease [81]. Follow up by FISH is not generally recommended due to the very limited sensitivity of this technique (Table 2), but it may play a role if quality controlled molecular monitoring by RT-qPCR or RT-dPCR is not available e.g., in resource-limited settings, or for cases expressing atypical *BCR::ABL1* variants. Similarly, the use of qualitative RT-PCR analysis is not recommended but may be of some value in resource-limited settings to rule out relapse or non-adherence [82]. Finally, conventional cytogenetics is no longer the sole test required to confirm complete cytogenetic remission if validated RT-qPCR testing is available (see below), but it may be useful in cases with overt relapse and /or evidence of disease progression to detect the emergence of ACAs.

**Table 2:** Recommended tests for monitoring or investigating CML patients on treatment.

	ROUTINE INVESTIGATIONS ON THERAPY	EXPERIMENTAL/CLINICAL TRIALS
**Interphase FISH**	• **Not recommended** May be useful for monitoring response if quality-controlled RT-qPCR not available, including patients with atypical *BCR::ABL1* fusions. Cannot be used to define MMR or DMR.	• **Not recommended**
**Qualitative RT-PCR**	• **Not recommended** Very limited value for monitoring response to treatment.	• **Not recommended**
**Cytogenetics**	• **Suggested at overt hematological relapse, failure according to ELN, or suspected/overt disease progression** • **Considered in cases in remission but abnormal blood counts** Only technique that can detect prognostically-significant ACAs acquired during the course of disease, and chromosome abnormalities in Ph-negative cells.	• **Should be considered**
**Quantitative RT-qPCR or RT-dPCR**	• **Mandatory for routine molecular monitoring** Only technique(s) that can quantify disease levels over full response range specific by ELN and other clinical recommendations, including DMR	• **Mandatory**
**NGS panel for myeloid and lymphoid genes**	• **Suggested at overt hematological relapse and suspected or overt disease progression** May be helpful to confirm progression and occasionally identify potential therapeutic targets	• **Recommended** Panel analysis during remission useful to determine if variants detected pre-treatment are somatic/germline/clonal hematopoiesis
***BCR::ABL1*** **TKD mutations**	• **Strongly recommended in cases who fail to reach defined ELN milestones or loss of MMR on TKI therapy** Informs subsequent treatment in many cases	• **Strongly recommended**

### The International Scale for *BCR::ABL1* measurement

**Fig. 4:**
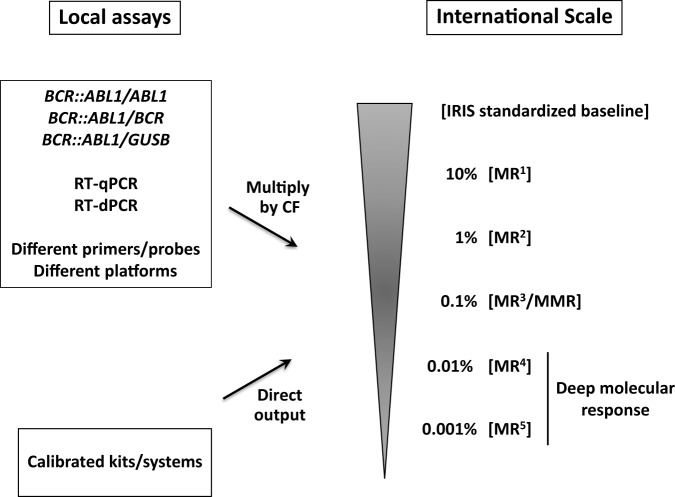
The International Scale for *BCR::ABL1* mRNA measurement. IS values are expressed as percentages and/or molecular response (MR) levels relative to the IRIS standardized baseline. Testing laboratories use either (i) RT-qPCR or RT-dPCR to measure the ratio of *BCR::ABL1* mRNA to that of a reference gene (*ABL1*, *GUSB*, *BCR*) and convert to the IS by multiplying the raw result by a laboratory-specific conversion factors (CF), or (ii) a validated kit/system that directly outputs results on the IS.

In addition to *BCR::ABL1*, the number of *ABL1*, *GUSB* or *BCR* reference gene transcripts is determined for all samples to gauge their quality, and to estimate the upper level of MRD in samples for which *BCR::ABL1* mRNA is not detected [83]. Any of the 3 reference genes is acceptable for routine analysis, but *ABL1* is used in the great majority of testing laboratories. Other reference genes are not recommended because they have not been calibrated to the International Scale (IS) for *BCR::ABL1* measurement [84]. For e13a2 and/or e14a2 cases, the ratio of *BCR::ABL1*/reference gene expression levels should be reported on the IS, whether derived by RT-qPCR or RT-dPCR. The IS defines MRD levels as a percentage relative to the standardized baseline used in the IRIS trial (Fig. 4) and not to pre-treatment levels for each patient (as is customary for MRD assessment in other hematological malignancies) [83, 85]. If *BCR::ABL1* is undetected (molecularly undetectable leukemia), the number of reference gene transcripts in the same volume of cDNA used to test for *BCR::ABL1* indicates the sensitivity for that particular sample (Table 3). Key points on the IS are indicated as molecular response (MR) levels, for example a BCR::ABL1^IS^ value of ≤0.01% is a ≥ 4-log reduction from the standardized baseline and abbreviated as MR^4^. A level of ≤1% BCR::ABL1^IS^ (MR^2^) is broadly equivalent to complete cytogenetic remission [86]. Deep molecular response (DMR) is usually defined as MR^4^ or deeper, irrespective of whether *BCR::ABL1* is detected or not. Terms such as ‘complete molecular response’ or 0% BCR::ABL1^IS^ should not be used because the interpretation of negative results is only possible if the sensitivity for the sample in question is known, as indicated by the number of reference gene transcripts (which should be included on all clinical reports). Failure to detect *BCR::ABL1* for a patient with MRD may simply be related to a technical issue such as degraded RNA. For example, undetectable *BCR::ABL1* mRNA with 45,000 *ABL1* reference gene transcripts indicates MR^4.5^, but undetectable *BCR::ABL1* mRNA with <10,000 *ABL1* reference gene transcripts is not evaluable for DMR and should be considered as a technical failure (Table 3).

**Table 3:** Summary of reference gene numbers required for scoring deep molecular response.

	MR^4^	MR^4.5^	MR^5^
Minimum sum of reference gene transcripts irrespective of whether *BCR::ABL1* is detected or not^a^	10,000 *ABL1* 24,000 *GUSB*	32,000 *ABL1* 77,000 *GUSB*	100,000 *ABL1* 240,000 *GUSB*
BCR::ABL1^IS^ level for positive samples^b^	≤0.01%	≤0.0032%	≤0.001%

^a^numbers of reference gene transcripts in same volume of cDNA that is tested for *BCR::ABL1*. The minimum number of reference gene transcripts in any individual replicate should be 10,000 *ABL1* or 24,000 *GUSB* (equivalent levels for *BCR* have not been determined).

^b^provided that the minimum reference gene copy numbers in the row above are fulfilled.

Testing laboratories using RT-qPCR or RT-dPCR generally express results on the IS by using (i) laboratory-specific conversion factors (CFs) to the IS derived by sample exchange with an established reference laboratory [85, 87, 88], (ii) laboratory-specific CFs derived from secondary reference reagents calibrated to the 1st World Health Organisation (WHO) International Genetic Reference Panel for the quantitation of *BCR::ABL1* [84, 89] or (iii) validated diagnostic kits or devices calibrated to the WHO panel. Technical guidelines have defined in detail how to derive IS values from raw RT-qPCR (or RT-dPCR) data, including sample quality criteria, how to score replicate reactions and what to do if *BCR::ABL1* mRNA is not detected [90, 91]. The frequency of monitoring should follow, resources permitting, that specified by the current version of the European LeukemiaNet (ELN) clinical guidelines.

### Technical considerations

To enable assessment of DMR, it is important that laboratories use optimised tests that are able to detect MR^4.5^ in most clinical samples. This requires the ability to detect small numbers of *BCR::ABL1* mRNA targets as well as good quality RNA/cDNA and consistent criteria to distinguish between low-level positive and undetectable disease, as described in detail elsewhere [90, 91]. Determination of the LoD must be performed with clinical samples; estimates of sensitivity derived solely from cell line mixtures are meaningless and should not be used or quoted on clinical reports. Optimization of test performance to achieve the desired LoD may be necessary, but attention should also be paid to the limit of blank (LoB), i.e., the background false positive rate, which may compromise accurate determination of DMR [91]. Use of automated, sensitive RT-qPCR systems for measuring *BCR::ABL1* on the IS, such as the Cepheid GeneXpert Ultra, or IS-calibrated RT-qPCR/RT-dPCR kits validated to detect DMR, e.g. [81, 92, 93], is often considered preferable to the complex and expensive process of in-house method development, validation and calibration to the IS. The GeneXpert system is particularly useful in resource-poor settings [9, 80, 94] but is also widely used in developed countries [95, 96].

Anticoagulated (not heparin-based) peripheral blood samples should be of adequate volume (e.g., ≥4-5mls) and delivered to the testing laboratory as soon as possible, ideally within 24 hours and no later than 48 hours. RNA should be extracted from total leukocytes, not mononuclear cells, or any other cell fraction. In resource-poor settings with no local facilities to perform MRD analysis, dried blood spots may be sent by regular mail at ambient temperature to a suitable reference laboratory, although the test sensitivity is not as good as that performed on fresh samples [97].

Ongoing performance evaluation (internal quality control; IQC) is required to detect assay drift and variability, e.g. by frequent analysis of standards designed to represent high and low levels of disease, ideally on every run in conjunction with the results feeding into run acceptability criteria [91, 98, 99]. For laboratory-developed tests, CFs may need to be revalidated (e.g. annually) and potentially changed at appropriate intervals in the context of IQC data, particularly if there is any change in methodology or equipment. CF revalidation may be performed by sample exchange with a reference laboratory, calibrated secondary reagents, kits or systems, or by using stored samples with known IS values. Definitions of optimal and satisfactory variation in CF values over time have been described [87, 91]. Unsurprisingly, laboratories with unstable assays tend to perform poorly, with CFs that often fail to validate [91]. In addition to a focus on maximizing assay stability, it is important to ensure that both *BCR::ABL1* and the chosen reference gene are amplified with comparable efficiencies, a parameter that is influenced in part by the amount of input material [89]. Differences in RT-qPCR amplification efficiency may result in distortion of the estimated disease burden at both high and low levels. Amplification efficiencies can easily be checked over time using plasmids that harbor both *BCR::ABL1* and reference gene targets, e.g. ERM-AD623 [91, 100].

As an endpoint measurement, RT-dPCR is less susceptible to differences in amplification efficiency compared to RT-qPCR, although the very wide dynamic range required for MRD assessment is a challenge for any technology. Although RT-dPCR is not intrinsically more sensitive than RT-qPCR [81, 101], the relative ease with which this technology can be used to measure very low levels of disease, coupled with the ability to readily test a larger amount of cDNA with multiple replicates, have resulted in several publications describing the utility of dPCR for patients in DMR before or after stopping treatment [102–105]. As noted above, a validated IS-calibrated diagnostic dPCR kit is available [81, 93] but in general dPCR approaches and analysis remain largely unstandardized and the degree of variation in results between centers is unclear.

Tests should be fully validated for clinical reporting, e.g., as described [106]. Laboratories should measure the variability of their test and include this information in clinical reports so treating physicians are informed of the uncertainty of measurement (UoM) at key clinical decision points, e.g., 10% BCR::ABL1^IS^ and 0.1% BCR::ABL1^IS^. In an international survey of laboratory performance, the mean standard deviation in *BCR::ABL1* measurement was 0.2 log, or approximately 1.6 fold on a linear scale, with a greater degree of variation at lower levels of disease [89]. For example, at 0.1% BCR::ABL1^IS^ (MR^3^, also known as major molecular response or MMR) this degree of variation equates to 0.063–0.16% which may be considered as the UoM at this level. Other recommendations for reporting MRD results and further details on test variability at high and low levels have been described in detail elsewhere [91, 107].

### Velocity of disease reduction on treatment

Analysis of the relative clone size pretreatment by RT-qPCR has revealed significant variation between patients. Furthermore, the initial velocity of disease reduction on therapy, usually measured as the halving time, has prognostic value for both early and late response, including TFR [40–42]. Focusing on patients who do not achieve the early molecular response milestone (≤10% BCR::ABL1^IS^ at 3 months on treatment), the rate of reduction of *BCR::ABL1* mRNA from baseline levels can help to distinguish patients who are destined to do relatively well with no change in treatment from those who may require an alternative TKI [40–42].

Standardized measurement of pretreatment disease levels by RT-qPCR (or RT-dPCR), however, is problematic due to the different properties of the 3 reference genes that have become established for CML MRD analysis. These differences are not relevant for standard MRD assessments, but they result in reference gene-specific distortions at high disease burdens. Most primer/probe sets for *ABL1* actually amplify *ABL1* plus *BCR::ABL1* and thus the maximum value of *BCR::ABL1*/*ABL1* reaches a plateau. Similarly, the expression levels of both unrearranged *BCR* alleles are measured when the CML clone is small relative to normal cells, but this reduces to the single unrearranged allele when the clone size is high. The net result of the use of different reference genes is that the IS begins to break down above 10% BCR::ABL1^IS^. Only *GUSB* is completely independent of *BCR::ABL1*, however a recent international comparison revealed relatively high and currently unexplained variability of MRD results between laboratories using *GUSB* [91]. Although the issues with *ABL1* and *BCR* could in principle be easily corrected mathematically, this does not work well in practice due to differences in amplification efficiencies for *BCR::ABL1* and reference genes between laboratories [91]. At the current time, therefore, a standardized approach to defining the rate of reduction of *BCR::ABL1* mRNA from baseline levels is very challenging and specific, generally applicable recommendations cannot be made. Consequently, this remains a research tool. In general, however, the use of *GUSB* or *BCR* as reference genes is expected to be of greater value than *ABL1* for assessment of early response kinetics although this remains to be proven in prospective studies.

### MRD for patients with atypical *BCR::ABL1* transcripts

Identification of cases who express atypical *BCR::ABL1* variants as the predominant isoform prior to treatment is important to enable appropriate follow up MRD analysis. Atypical fusions are either not detected by standard RT-qPCR or RT-dPCR configurations, or they are detected very inefficiently. Thus, patients may be incorrectly assessed as very good responders to treatment if the presence of an atypical variant is not recognised. MRD analysis for patients with atypical *BCR::ABL1* variants needs to be performed with bespoke, validated RT-qPCR or RT-dPCR tests; results cannot be expressed on the IS and thus standard time-dependent molecular milestones and guidelines for treatment discontinuation cannot be applied. However, the general tempo and depth of response can be assessed in these cases as individual molecular response (IMR) levels in relation to baseline (pre-treatment) *BCR::ABL1* expression prior to therapy [108]. Since these fusions are rare, monitoring is most appropriately performed in a suitably qualified reference laboratory, preferably using *GUSB* as a reference gene to help ensure the linearity of measurements at high disease levels.

Although the ELN 2020 recommendations specify that TKI discontinuation to achieve TFR should only be considered for patients expressing standard e13a2 and/or e14a2 *BCR::ABL1*, there is a growing recognition that cases expressing atypical *BCR::ABL1* variants may also be candidates to stop treatment. From a technical perspective, we suggest that persistent molecularly undetectable disease for >2 years and ≥4 log reduction from baseline levels might be used to consider eligibility to stop treatment alongside other clinical requirements. The test employed must have been validated to detect low disease burdens, and evaluable patient samples must have ≥10,000 *ABL1* and/or ≥24,000 *GUSB* transcripts, and ideally ≥32,000 *ABL1* and/or ≥77,000 *GUSB* transcripts (Table 3). As for standard e13a2/e14a2 cases, frequent monitoring after cessation is very important to detect molecular relapse. FISH testing, even with the analysis of hundreds of cells, is generally considered as insufficiently sensitive to guide treatment cessation. We recognise, however, that efforts need to be made to improve the general availability of high-quality molecular monitoring for atypical *BCR::ABL1* variants.

### Clinical associations with common *BCR::ABL1* isoforms

Several studies have shown that patients expressing e13a2 *BCR::ABL1* have inferior molecular responses at various timepoints after starting imatinib compared to those expressing e14a2. Most kits and LDTs use an RT-qPCR design that employs a pair of primers and a single probe to detect both e13a2 and e14a2 *BCR::ABL1* cDNA without distinguishing between them. Recent evidence indicates that at least part of the observed differences in molecular response are explained by small differences in PCR amplification efficiency between the two transcript types, with the larger e14a2 amplifying less efficiently and thereby giving the appearance of a superior response [109–111]. Results from transcript-specific assays may also be influenced by differences in amplicon size. From a technical perspective, these differences may be overcome by using RT-dPCR, which is inherently more tolerant to factors that influence RT-qPCR efficiency [111]. The differences observed by RT-qPCR, however, are small compared to the natural variation in test results, do not translate into inferior outcomes [112] and are believed to have minimal effect on the management of individual patients [110]. In addition, some studies have found that *BCR::ABL1* transcript type correlates with the success of attempted achievement of TFR, specifically e14a2 cases have a higher probability of remaining in remission[113, 114], but the effect is not considered strong enough to influence clinical management.

### Other approaches to MRD testing

Theoretically, DNA-based MRD analysis has an advantage over RNA analysis in that results are intrinsically easier to standardize and can be readily related to cell numbers. For CML, however, the genomic breakpoints need to be characterized for each individual and patient-specific qPCR or dPCR tests designed and validated, which is time consuming and expensive. Although DNA-based tests have provided useful insights into the biology of TFR [115] and may help to predict TFR outcomes [105] it seems unlikely that this approach will become cost effective for routine use. Other areas of development include single cell genomic analysis, for which there has been rapid technological progress in recent years. This technology has the potential to measure MRD over a wide dynamic range and provide information on the cellular identity of residual *BCR::ABL1* positive cells [79], but is probably many years away from routine clinical use.

### Recommendations:

- MRD analysis for CML should use *ABL1*, *BCR* or *GUSB* as internal reference genes. RT-dPCR is an acceptable alternative to RT-qPCR and may offer some technical advantages, particularly for assessment of very low-level disease.
- MRD results using RT-qPCR or RT-dPCR should be expressed on the International Scale for *BCR::ABL1* measurement for e13a2/e14a2 *BCR::ABL1* patients.
- For assessment of deep molecular response, tests should be optimised to achieve a limit of detection of MR^4.5^ or better using clinical samples, without compromising the limit of blank (background false positive rate).
- Ongoing internal quality control is essential to monitor assay stability over time.
- Patients expressing atypical *BCR::ABL1* fusions should be monitored by bespoke RT-qPCR or RT-dPCR assays and results expressed as individual molecular responses compared to baseline levels.
- FISH is not recommended as this technique has very limited sensitivity compared to molecular monitoring but it may be useful if molecular tests for atypical fusions are not available.

## BCR::ABL1 mutations and mechanisms of resistance to TKIs

### Background

**Fig. 5:**
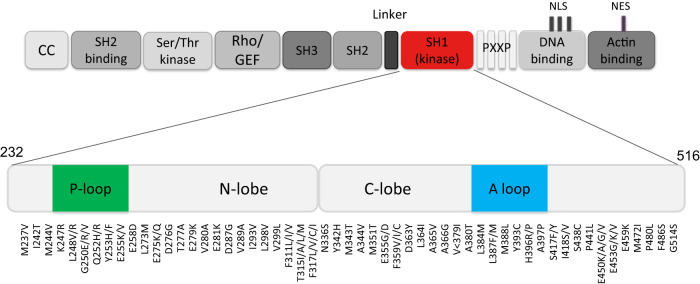
BCR::ABL1 TKD mutations. Map of ABL1 indicating the mutations reported in the literature to be associated with resistance to ATP-competitive TKIs (imatinib, dasatinib, nilotinib, bosutinib and ponatinib).

Among the variety of resistance mechanisms that may underlie a non-optimal response to TKI therapy in CML patients, secondary point mutations in within the region of *BCR::ABL1* encoding the TKD are the only ones that are clinically actionable. Mutations may alter critical contact residues between BCR::ABL1 and the inhibitor, or induce conformational changes that result in reduced TKI binding affinity. Rational development of 2G, 3G and 4G TKIs has offered alternative therapeutic options for patients with unsatisfactory responses, including those positive for *BCR::ABL1* mutations [116]. Over the years, integration of in vitro measurement of the half-maximal inhibitory concentration or IC_50_ (generally determined as the concentration necessary to inhibit by 50% the proliferation of BaF3 cells engineered to express a specific BCR::ABL1 mutant) and in vivo observation of mutations selected in patients with non-responsive disease on a given TKI has helped to understand which imatinib-resistant mutations (and which newly described mutations) may confer resistance to dasatinib, nilotinib, bosutinib, ponatinib (Fig. 5). More recently, in vitro IC_50_ data have also been generated for several combinations of mutations (‘compound mutations’), i.e. two mutations *in cis* on the same BCR::ABL1 molecule), that may be observed in a proportion of patients, mainly advanced phase patients who have failed ≥2 lines of TKI therapy [117–121]. Based on these data, it appears that many compound mutations might confer resistance to 2G TKIs and some appear to confer resistance to ponatinib, specifically those including the T315I variant. However, in vivo evidence is still limited.

**Fig. 6:**
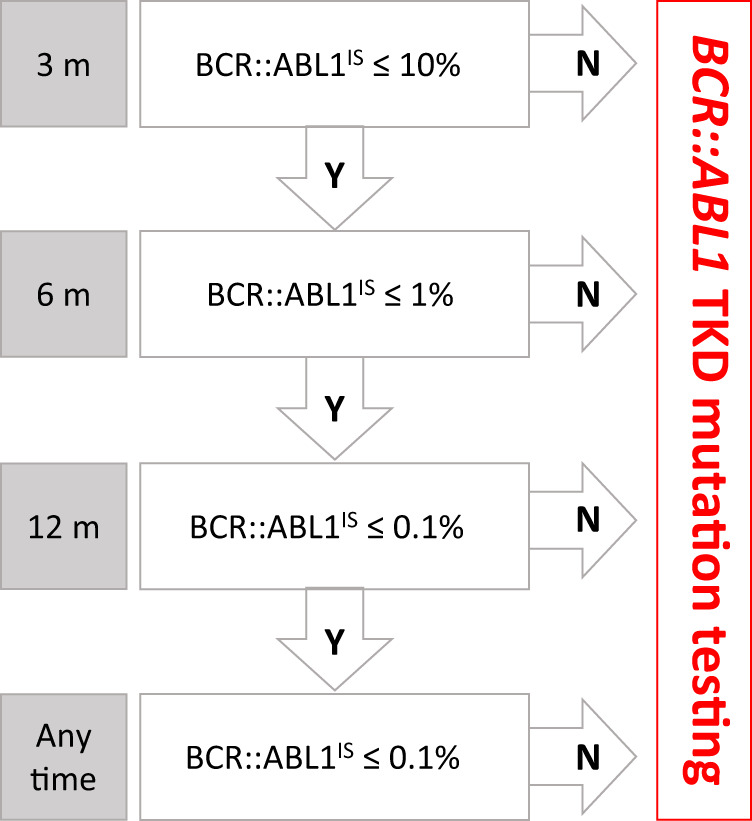
Practical algorithm indicating when *BCR::ABL1* TKD mutation testing should be performed based on *BCR::ABL1* transcript levels at each timepoint during therapy. For example, at 6 months on treatment a patient who has not achieved (N) ≤ 1% BCR::ABL1^IS^ should be considered for *BCR::ABL1* TKD testing. If ≤ 1% BCR::ABL1^IS^ has been achieved (Y) then TKD testing is not required and the patient should be reassessed at 12 months. TKD mutation testing is not generally recommended for any patient with ≤ 0.1% BCR::ABL1^IS^, in part because the sensitivity of detection is low and amplification of the large fragment required for *BCR::ABL1*-specific mutation detection may be challenging.

The clinical value of *BCR::ABL1* TKD mutations testing at relevant timepoints on treatment (Fig. 6) is widely recognized [122]. Positivity for a TKI-resistant mutation should usually trigger a change of therapy, and detection of specific mutations helps exclude TKIs that are unlikely to be effective. Specific recommendations about when to perform *BCR::ABL1* TKD mutation testing, how to perform it and how to interpret and translate results into clinical decisions were formulated by a panel of experts appointed by the ELN in 2011 [122]. However, definitions of non-optimal response, to which indications for mutations testing were anchored, have since changed [116]. In addition, more data have been consolidated and are now available on the mutation vulnerabilities of 2G and 3G TKIs. Most importantly, technologies for mutation testing have greatly evolved over the past decade, and NGS and dPCR are now supplanting the role of Sanger sequencing as the gold standard for *BCR::ABL1* TKD mutation testing [123–125]. Retrospective and prospective studies with NGS have shown that its greater sensitivity may provide a more accurate picture of mutation status and may pick emerging TKI-resistant mutations in a timelier manner [123, 126–130].

### How to perform BCR::ABL1 TKD mutation testing

By convention, the numbering for BCR::ABL1 resistance-associated mutations is made by reference to the ABL1 isoform a (i.e. encoded by the normal *ABL1* transcript that includes alternative first exon 1a), for which the Matched Annotation from the NCBI and the EMBL-EBI (MANE) select versions [131] at the time of writing are: protein, NP_005148.2; mRNA: NM_005157.6; Ensembl: ENST00000318560.6. It is important to avoid the use of the ABL1 isoform b (the isoform encoded by the transcript that includes alternative first exon 1b) when reporting BCR::ABL1 TKD mutations since the numbering of residues would be shifted by 19 amino acids, (e.g., the T315I mutation in isoform 1a would be T334I in isoform 1b). Of note, however, ABL1 isoform b numbering is commonly used in biochemical publications focusing on ABL1 structure, regulation and signalling outside the field of CML. Clinical reports should include full HGVS recommended nomenclature (with a DNA/cDNA level description, application of the preferable three letter amino acid code at the protein level and accompanying appropriate reference sequence) to ensure an unequivocal description of variants. However, much of the current clinical guidance and associated literature uses an abbreviated single letter protein variant description, which should also be clearly indicated on the report. For example, the variant NM_005157.6:c.944 C > T p.Thr315Ile (detected from cDNA) or NM_005157.6:c.944 C > T p.(Thr315Ile) (detected from genomic DNA) is generally abbreviated to T315I. Simplified notation is used for all variants henceforth.

The TKD is a relatively large region encompassing residues 232-516 (O. Hantschel, personal communication). Multiple mutations between amino acids 217 and 514 have been identified in imatinib-resistant patients, with some falling in key functional regions (e.g., G250E, Y253H, E255K/V in the P-loop; T315I and F317L at TKI contact residues; M351T and F359V in the SH2 contact region; L387M and H396R in the activation loop) detected more frequently than others. Mutants acknowledged to be resistant to 2G TKIs are much fewer (Table 4). Several T315I-inclusive compound mutations have been reported in ponatinib-resistant patients (T315I/E255K, T315I/E255V, T315I/F359V, T315I/G250E; T315I/M351T) [120, 132] as well as two single point mutations, T315M and T315L, that emerged from pre-existing T315I clones following a further nucleotide change [127, 132, 133]. The allosteric inhibitor asciminib binds to the myristoyl-binding pocket whereas all other TKIs target the ATP-binding pocket. Consequently, the mutational spectrum associated with asciminib resistance reported to date is largely distinct from other TKIs, e.g., P223S, A337V, P465S, V468F and I502L are specifically associated with asciminib resistance [134, 135] as well as other mutations reported to have been selected in patients who have failed asciminib in published clinical trials [136–139] (Table 4). Some of these mutations (e.g. P223S) are outside the TKD, but still in areas covered by most amplification strategies currently employed to search for mutations in ATP-competitive inhibitor-resistant patients. It is likely that the full spectrum of mutations leading to asciminib resistance remains to be documented. Indeed, the imatinib- and nilotinib-resistant F359V mutant has been predicted in vitro, and seemingly confirmed in vivo, to be poorly sensitive to asciminib [137]. Finally, compound mutations involving T315I have also been reported in asciminib-resistant patients (T315I/E255K, T315I/F359I, T315I/E355G, T315I/M351T, T315I/E453Q) [140].

**Table 4:** Mutations that have been consistently reported in the literature to confer resistance to 2G TKIs, ponatinib and asciminib^a^.

**Mutations conferring resistance to dasatinib**	V299L, T315I/A, F317L/V/I/C
**Mutations conferring resistance to nilotinib**	Y253H, E255K/V, T315I, F359V/I/C
**Mutations conferring resistance to bosutinib**	E255K, V299L, T315I
**Mutations conferring resistance to ponatinib**	T315M/L
**Mutations conferring resistance to asciminib**^b^	G109D, Y115N, M244V, V289I, A337V/T, E355G, F359V, E462K, G463D/S, P465S, V468F, S501R, I502L

^a^Combinations of mutations *in cis* on the same *BCR::ABL1* molecule (compound mutations) are likely to display peculiar resistance profiles, different to those the individual mutants would display if present in trans on different molecules (i.e., independent clones). However, the in vivo data needed to complement in vitro IC_50_ predictions are scarce, so precise indications cannot be formulated. It is likely that the great majority of compound mutations will be resistant to imatinib and 2G TKIs. Some T315I-inclusive compound mutations have so far been reported in ponatinib-resistant patients [120, 132] and also in asciminib-resistant patients [140].

^b^For asciminib-resistant mutations, the list is provisional and includes mutations within and outside the TKD reported to have been selected in patients who failed asciminib in published clinical trials [136–139]. These patients had been pretreated with multiple TKIs and further data will be needed to compile a more robust list.

**Fig. 7:**
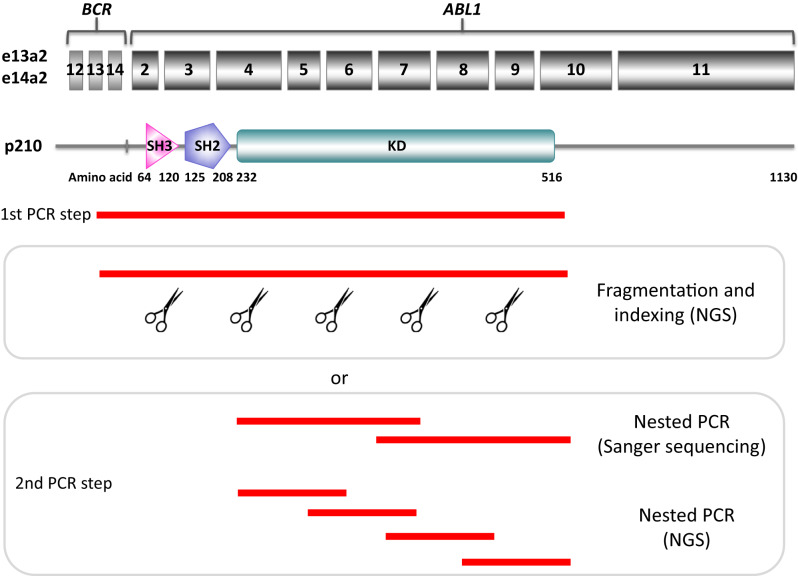
Schematic representation of the strategies commonly employed to amplify and sequence the TKD, either by Sanger sequencing or by NGS. After reverse transcription of RNA to cDNA, a first step of amplification is performed using a forward primer mapping to *BCR* sequences close to the breakpoint (usually in exon 12 or 13, to amplify both e13- and e14- transcript variants) and a reverse primer mapping to *ABL1* sequences immediately 3’ prime of the sequencing encoding the TKD (usually exon 10). The first amplicon may then be fragmented, indexed and sequenced by NGS or subjected to a second step of amplification using a series of internal primer pairs generating amplicons of suitable length for either Sanger sequencing or NGS.

Only a sequencing approach may offer a detailed snapshot of TKD mutation status. For this reason, Sanger sequencing was recommended in 2011 as the gold standard for *BCR::ABL1* TKD mutation screening [122]. Mutation testing by Sanger sequencing is relatively fast and easy, but it has a relatively poor LoD, generally estimated to be a VAF of around 15–20% - i.e., 15–20 mutant transcripts per 100 total *BCR::ABL1* transcripts (although depending on sequence context and quality, mutations with a VAF of around 10% may be detected in some instances). The use of targeted NGS has been explored in several retrospective and prospective studies. NGS presents both advantages and disadvantages. On one hand, its lower LoD (3–5% VAF for standard NGS; ≤0.5% for error corrected NGS) allows more accurate assessment of mutation status, including early detection of emerging mutations that have not yet reached the threshold for Sanger sequencing detection. Moreover, NGS theoretically enables clonal analysis, hence straightforward identification of compound mutations. However, two caveats exist in this context: the spectrum of mutation pairs for which the *in cis* versus in trans configuration can be assessed is dependent upon a) the sequencing chemistry and read length, with longer reads preferred, and b) the phenomenon of PCR-mediated recombination may impair reliable detection of low-level compound mutations [141]. Many protocols employed nested or hemi-nested RT-PCR to sequence the region encoding the ABL1 TKD from the *BCR::ABL1* fusion and not the normal unrearranged *ABL1* allele (Fig. 7). Whilst this approach provides excellent specificity, it may result in a higher level of technical artefacts, and thus a single amplification protocol is preferred whenever possible.

Widespread implementation of NGS-based approaches of *BCR::ABL1* TKD mutation testing needs to address some challenges: i) no commercial kits are available, which poses the need to establish and internally validate an LDT; ii) the throughput of the instruments mandates batching samples in each sequencing run. The need to balance cost-effectiveness and turnaround time makes NGS analysis practically feasible only if samples are centralized in reference laboratories. Implementation of NGS-based mutation detection is particularly challenging in developing countries, where new accessible technologies are required.

Alternative methods have been described that offer greater sensitivity as compared to Sanger sequencing. However, they rely on the use of allele-specific primers or probes, hence they can be implemented only for a limited number of mutations of interest. A multiplexed approach of primer extension coupled with mass spectrometry-based identification of the extended nucleotides (Sequenom MassArray) has been described that allows scanning for 31 different mutations (imatinib- and 2G TKI-resistant) [142, 143]. The MassArray, however, is an expensive, high-throughput platform and arguably more suited to large-scale research studies rather than to routine diagnostic testing. More recently, a RT-dPCR-based approach for detection of 16 nucleotide substitutions associated with mutations conferring resistance to 2G TKIs (that is, the mutations more readily impacting on TKI selection once the decision to switch the patient for unsatisfactory response has been taken) has been described [125]. If optimized, RT-dPCR is more accurate and sensitive than NGS; moreover, it is easier and faster. However, the possibility to implement it for a panel of predefined sequence variants limits its use to specific actionable mutations. Thus, its utility is limited to patients receiving or being considered for a specific TKI for which assays for known resistance-associated mutations are available. It has to be borne in mind, however, that any secondary *BCR::ABL1* mutation might be considered as a measure of the degree of genetic instability, thus positivity for any mutation might identify higher-risk patients requiring more careful monitoring. In this context, given the wide number of mutations associated with imatinib resistance, a targeted dPCR screen may be of limited value in the setting of a warning response to imatinib, where positivity for an imatinib-resistant mutation would be a decisive indication for TKI switch.

Set against these considerations is the fact that the clinical utility of TKD mutation detection by Sanger sequencing has been firmly established in multiple retrospective and prospective studies. By contrast, not all low-level mutations identified by NGS necessarily herald relapse. Whatever the method used to detect the nucleotide substitutions, it is generally acknowledged that the best input nucleic acid for *BCR::ABL1* TKD mutation testing is cDNA rather than genomic DNA. The former enables (pre-)amplification and specific analysis of *BCR::ABL1* fusion transcripts. In contrast by using DNA both *ABL1* and *BCR::ABL1* alleles would be sequenced—which would usually result in an inferior LoD because the number of *BCR::ABL1* alleles (present only in leukemic cells) are far fewer than the number of *ABL1* alleles (present in both leukemic and normal cells). Use of high sensitivity techniques like error-corrected NGS or digital PCR might theoretically enable this issue to be circumvented [144], and indeed the utility of DNA-based targeted dPCR for T315I has been described in patients with advanced disease [145], but a systematic comparison would be needed before generally endorsing DNA-based strategies.

Sequencing chemistries and technologies are continuously evolving, and third generation sequencers that enable real time long (several kilobases) sequencing reads are beginning to enter routine practice. The use of long read Nanopore and PacBio sequencing has been reported for *BCR::ABL1* TKD screening [146, 147], but it is currently unclear if these or other third generation sequencing chemistries will ultimately supplant current approaches.

### Recommendations:

- Targeted NGS, whenever available or accessible, is the recommended method for *BCR::ABL1* TKD mutation testing, provided that a reliable and validated assay has been implemented.
- Mutation screening should only be performed for cases with *BCR::ABL1* mRNA levels ≥0.1% IS, i.e., ELN-defined failure or warning.
- The recommended input material is RNA from peripheral blood or bone marrow leukocytes. Analysis of genomic DNA is not recommended for routine analysis.
- Digital PCR may be considered as a first-level screening test for detection of specific mutations of interest when (rapid) TKI switch is needed.
- The use of Sanger sequencing is acceptable whenever NGS is not available or accessible, and does not represent an inappropriate management of patients.
- The minimal recommended region to be covered in a broad, untargeted mutation screen for 1st, 2nd and 3rd generation TKIs should focus on the sequence encoding the TKD (residues 232 to 516; Fig. 5). However, for patients resistant to asciminib, mutation screening should be extended to include relevant regions outside the TKD (Table 4).

### When to perform *BCR::ABL1* TKD mutation testing

It is generally acknowledged that mutations surface as a result of the selective pressure imposed by TKI therapy. At diagnosis, before the start of any TKI treatment, mutations—if any—would be undetectable with the currently available methods. This is likely to hold true both for CP and for the very rare cases who are diagnosed in BP, although data for the latter are extremely scarce [148]. On treatment, the role of *BCR::ABL1* TKD mutation testing is to support clinical decision making whenever response is not satisfactory. The ELN recommendations distinguish unsatisfactory responses into failures and warnings. Failure usually mandates a change of therapy, although the possibility of non-compliance should also be investigated. Warning is a grey area where continuation or change is equally possible, based on careful evaluation of patient’s characteristics, comorbidities and tolerance as well as the kinetics of molecular response. According to the ELN clinical recommendations, detection of a TKI-resistant mutation is among the definitions of failure [116], and thus *BCR::ABL1* TKD mutation testing in warning cases tilts the balance towards TKI switch. Definitions of failure and warning responses are currently available for patients on first- and second-line treatment. Loss of MMR whilst under TKI therapy is an important failure criterion which is relatively frequently associated with acquisition of a TKI-resistant mutation. This should be distinguished from loss of MMR in the context of a TKI discontinuation attempt, in which case mutation testing is not warranted as patients are known to respond well to their previous TKI. The frequency of TKI-resistant mutations by Sanger sequencing in failures and warning cases is estimated to be between 20–35% and 10–20%, respectively (with ranges depending on TKI, line of therapy and disease phase). The frequency of TKI-resistant mutations by NGS in failure and warning cases has been reported to be 40-50% and 30-40%, respectively [130].

### Recommendations:

- *BCR::ABL1* TKD mutation testing is indicated:
  - In case of failure and warning to 1st or 2nd line TKI therapy according to current ELN clinical recommendations.
  - In case of relapse (BCR::ABL1^IS^ ≥ 0.1%) after allogeneic stem cell transplant if mutation was detectable prior to transplant.
- *BCR::ABL1* TKD mutation testing is not indicated:
  - at diagnosis, i.e., before start of any TKI treatment.
  - at failure after TFR (except for patients not achieving MMR within 3–6 months after restarting TKI therapy when TKD mutation testing is indicated).
  - In patients with BCR::ABL1^IS^ < 0.1%.
- In patients on third-line TKI therapy and beyond, *BCR::ABL1* TKD mutation testing should be considered when there is no improvement of response after 3–6 months of treatment, and is indicated if response worsens (e.g. ≥5-fold increase of BCR::ABL1^IS^with loss of MR^3^).
- Sequential monitoring of a positive *BCR::ABL1* TKD mutation testing result might be useful in case of low level mutations of unknown or doubtful clinical significance. Resampling should be performed after 1–3 months (depending on response level and kinetics).
- Sequential monitoring of a positive *BCR::ABL1* TKD mutation testing result might also be useful if there is no improvement in response based on standard *BCR::ABL1* transcript level monitoring after TKI switch.

### How to use *BCR::ABL1* TKD mutation results

Whether a positive mutation result should lead to an immediate change of treatment depends on the level of non-optimal response, on the type and level of detected mutation(s) and on clinical considerations regarding the therapeutic alternatives available for each individual patient.

Although reverse transcription and PCR sequence errors may always occur irrespective of the downstream method used to search for mutations and even with the use of high-fidelity enzymes, Sanger sequencing and digital PCR are less prone to false-positive results than NGS. Without a strategy of error correction (such as the use of unique molecular identifiers to ‘barcode’ individual molecules), NGS artifacts are not infrequent, particularly at lower VAFs and/or lower levels of total *BCR::ABL1*. Bioinformatic analysis of NGS data should address this issue, taking all the necessary measures to minimize the likelihood of errors (e.g., using strict thresholds for coverage, quality scores of base calls, etc).

Comprehensive databases of BCR::ABL1 TKD variants detected in TKI-resistant patients have not been developed, but IC_50_ data are available for many mutations. Importantly, however, the cellular IC_50_ values do not always correlate with clinical response, and thus published clinical response data, if available, should always be considered. The possibility should also be considered that some rare sequence variants might be benign polymorphisms [149] or that they might just be innocent bystander mutations and not necessarily play a causal role in the unsatisfactory response that triggered mutation testing. By NGS, more variants of unknown clinical significance may be detected. The vast majority of these are found at low levels, raising a series of questions as to whether (i) they represent true, minor mutant subclones that cooperate to give rise to resistance, (ii) they are the expression of true, minor mutant subclones that transiently appear but do not confer a selective advantage and become dominant, (iii) they are just artifacts. In general, low-level variants, e.g. <15% VAF, that are not recognized as being recurrent in CML require confirmation on an independent sample before being considered as potentially clinically actionable. True resistance-driving mutations would be expected to expand on sequential analysis without change of therapy.

By NGS, insertions and deletions most probably resulting from alternative/abnormal splicing patterns are also a frequent finding. The so called ‘35ins’ variant for example, is the retention of 35 intronic nucleotides at the junction between exon 8 and 9 [150–152]. By NGS, this insertion has been observed in a (often, but not always) minor proportion of *BCR::ABL1* transcripts in up to 70% of patients screened for mutations. By Sanger sequencing, its prevalence is much lower (1–2% of resistant patients) because of the inferior sensitivity of the method or because, even if present in >20% of the transcripts, overlap of sequences with and without the insertion in output chromatograms could be erroneously interpreted as background noise resulting from unincorporated fluorescent dyes. While initial reports had suggested a role for 35ins in TKI resistance, a subsequent functional study has convincingly demonstrated that the resulting truncated protein (the insertion introduces a stop codon after 10 intron-encoded residues) lacks kinase activity and does not show oncogenic properties [153]. Consequently, this variant should not be reported. Based on all the above considerations, the clinical interpretation of mutation testing results should always be performed by a multidisciplinary team involving both molecular pathologists/laboratory scientists and clinical experts in CML biology and treatment.

### Recommendations:

- Mutations detected by Sanger sequencing or detected in ≥15% of transcripts by NGS or dPCR, should trigger consideration of an immediate change of therapy if they are recognized to be resistant to the ongoing TKI.
- Mutations detected in <15% of transcripts by NGS or digital PCR should trigger consideration of a change of therapy if they are recognized to confer resistance to the ongoing TKI and an immediate TKI change is deemed preferable over a wait-and-watch approach, for example in the context of continually increasing BCR::ABL1^IS^ levels or suspected/overt disease progression.
- Mutations detected in <15% of transcripts that are not recognized as being recurrent in CML should be confirmed on an independent sample before being considered further. Mutations of unknown clinical relevance (i.e. never reported in the literature) should not trigger an immediate change of therapy unless the clinician deems the risks of continuing the ongoing TKI treatment to be much greater than the benefits (for example, in case of warning).
- The ‘35ins’ variant should not be reported as a TKI-resistant mutation.

### *BCR::ABL1*-independent mechanisms of relapse

Approximately half of patients who relapse do so with no evidence of a secondary *BCR::ABL1* TKD mutation. Some of these may not be biologically driven and instead relate to non-adherence to therapy, which may be caused by a variety of reasons [154]. Others acquire diverse secondary abnormalities, including secondary ACAs and known or novel fusion genes [66, 67, 155] but none of these are clinically targetable and thus no specific investigations are considered as mandatory. As noted above, however, a myeloid gene panel may occasionally identify potentially targetable abnormalities in cases with overt relapse and evidence of disease progression. Patients who have persistent thrombocytosis or other features suggestive of a myeloproliferative neoplasm (MPN) whilst in remission for CML should be tested for *JAK2* V617F and other MPN driver mutations. For reasons unknown, the co-occurrence of *BCR::ABL1* and *JAK2* V617F is significantly more common than would be expected by chance [156]. In general, there is no impact of clonal chromosome abnormalities in Ph-negative cells on long-term outcome but, as mentioned above, some cases with -7/7q may develop *BCR::ABL1*-negative MDS or a related myeloid neoplasm [23, 157].

## Data Sharing

Data sharing not applicable to this article as no datasets were generated or analysed during the current study.
